# Evaluation of bi-directional causal association between obstructive sleep apnoea syndrome and diabetic microangiopathy: a Mendelian randomization study

**DOI:** 10.3389/fcvm.2024.1340602

**Published:** 2024-05-09

**Authors:** Qianqian Liu, Xingyu Chang, Rongna Lian, Qi Chen, Jialei Wang, Songbo Fu

**Affiliations:** ^1^ Department of Endocrinology, First Hospital of Lanzhou University, Lanzhou, Gansu, China; ^2^ Gansu Provincial Endocrine Disease Clinical Medicine Research Center, Lanzhou, Gansu, China; ^3^ Department of Gynecology, Obstetrics and Gynecology Hospital, Fudan University, Shanghai, China; ^4^ Center of Gerontology and Geriatrics, West China Hospital, Sichuan University, Chengdu, China

**Keywords:** obstructive sleep apnea syndrome, diabetic microangiopathy, Mendelian randomization, forced vital capacity, forced expiratory volume in 1 s

## Abstract

**Background:**

The relationship between obstructive sleep apnea syndrome (OSAS) and diabetic microangiopathy remains controversial.

**Objective:**

This study aimed to use bidirectional two-sample Mendelian Randomization (MR) to assess the causal relationship between OSAS and diabetic microangiopathy.

**Methods:**

First, we used the Linkage Disequilibrium Score Regression(LDSC) analysis to assess the genetic correlation. Then, the bidirectional two-sample MR study was conducted in two stages: OSAS and lung function-related indicators (forced vital capacity (FVC) and forced expiratory volume in 1 s (FEV1)) were investigated as exposures, with diabetic microangiopathy as the outcome in the first stage, and genetic tools were used as proxy variables for OSAS and lung function-related measures in the second step. Genome-wide association study data came from the open GWAS database. We used Inverse-Variance Weighted (IVW), MR-Egger regression, Weighted median, Simple mode, and Weighted mode for effect estimation and pleiotropy testing. We also performed sensitivity analyses to test the robustness of the results. Furthermore, we performed multivariate and mediation MR analyses.

**Results:**

In the LDSC analysis, We found a genetic correlation between OSAS, FVC, FEV 1, and diabetic microangiopathy. In the MR analysis, based on IVW analysis, genetically predicted OSAS was positively correlated with the incidence of diabetic retinopathy (DR), diabetic kidney disease (DKD), and diabetic neuropathy (DN). In the subgroup analysis of DR, there was a significant causal relationship between OSAS and background diabetic retinopathy (BDR) and proliferative diabetic retinopathy (PDR). The reverse MR did not show a correlation between the incidence of diabetic microangiopathy and OSAS. Reduced FVC had a potential causal relationship with increased incidence of DR and PDR. Reduced FEV1 had a potential causal relationship with the increased incidence of BDR, PDR, and DKD. Multivariate MR analysis showed that the association between OSAS and diabetic microangiopathy remained significant after adjusting for confounding factors. However, we did not find the significant mediating factors.

**Conclusion:**

Our results suggest that OSAS may be a cause of the development of diabetic microangiopathy, and OSAS may also be associated with a high risk of diabetic microangiopathy, providing a reference for a better understanding of the prevention of diabetic microangiopathy.

## 1 Introduction

Diabetic microangiopathy is one of the major complications of diabetes, with over 4.59 billion adults worldwide having diabetes, and over a third developing diabetic microangiopathy (1–4). Diabetic microvascular complications include diabetic retinopathy (DR), diabetic kidney disease (DKD), and diabetic neuropathy (DN) (5). Among them, DR is the most common, with a prevalence of 35.4% (6) and DR can be divided into background diabetic retinopathy (BDR) and proliferative diabetic retinopathy (PDR). The overall health of diabetic patients is severely affected by microvascular complications, leading to various adverse health consequences. Studies have shown that the combination of DKD and hypoglycemic events results in a significantly increased risk of falls and fractures and significant challenges in performing daily tasks such as walking and housework (7, 8). Furthermore, their incidence of chronic and acute health events is also higher than the general population (9). Most critically, patients with diabetic microangiopathy also have a significantly increased risk of death from cardiovascular complications and renal failure (10). The early prevention and management of diabetic microangiopathy are therefore essential.

OSAS is widely recognized worldwide as a significant respiratory disorder, with an estimated prevalence of 5%–15% in the general population and positively correlated with age, showing a gradual growth trend (11). The primary characteristic of OSAS recurrent episodes of sleep-dependent apnea and reduced airflow. Persistent OSAS can have detrimental effects on respiratory function, which is typically quantified using forced vital capacity (FVC) and forced expiratory volume in 1 s (FEV1) values. More critically, this respiratory disorder is closely related to increased risks of hypertension, coronary heart disease, and heart failure (12).

Previous observational studies have found a close connection between OSAS and diabetic microangiopathy (13–18). However, due to potential confounding biases and reverse causality in observational studies, their causal relationship is still unclear and requires further research to fully understand the potential mechanisms and establish the relationship between these diseases.

MR uses single nucleotide polymorphisms (SNPs) as instrumental variables (IVs) to infer the causal relationship between two traits, treating genetic variations as a “natural” randomized controlled trial. Individuals are randomly assigned to different exposure levels throughout their lives, minimizing biases caused by confounding factors and reverse causality (19–21). Therefore, this study aims to use samples based on the GWAS database for MR analysis to explore the causal relationship between OSAS and diabetic microangiopathy, which may guide the prevention and treatment of diabetic microangiopathy.

## 2 Materials and methods

### 2.1 Study design

**Figure 1:**
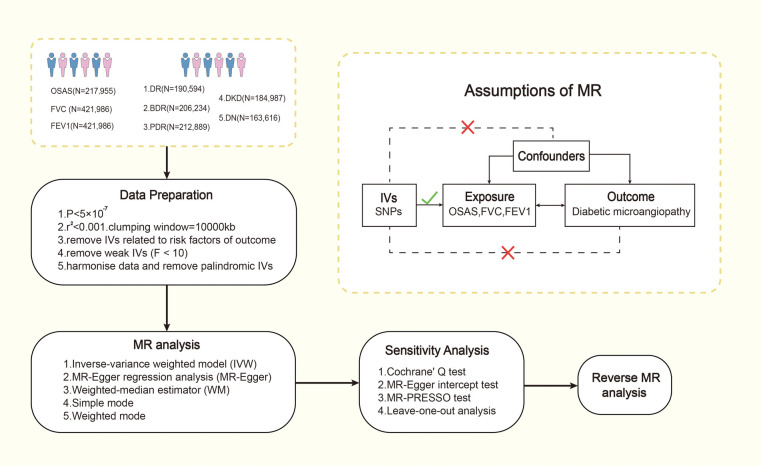
The process of Mendel randomization research. FVC, forced vital capacity; FEV1, forced expiratory volume in one second; IV, instrumental variable; MR-PRESSO, Mendelian randomization pleiotropy residual sum and outlier; MR, Mendelian randomization; OSAS, obstructive sleep apnea syndrome; SNP, single nucleotide polymorphism.

We employed a bidirectional two-sample MR study design, utilizing two-sample MR methods and varying GWAS summary-level datasets to elucidate the causal relationship and pathogenic direction between OSAS and lung function indicators (FVC, FEV1) with diabetic microvascular complications in European populations. This investigation was split into two phases. Initially, we probed whether OSAS had a causal relationship with diabetic microvascular complications. In the second stage, we evaluated if diabetic microangiopathy was associated with OSAS. The primary flow of our study is illustrated in Figure 1. We then conducted supplementary analyses, including a multivariate MR analysis to mitigate potential confounding factors and a mediation MR analysis to explore potential mediating factors. The MR design is based on three assumptions: (1) genetic variants are strongly associated with the exposure; (2) genetic variants are unrelated to other confounding factors; (3) genetic variants are associated with the outcomes solely through the investigated exposure. The association data of SNPs with OSAS and diabetic microangiopathy derive from recently published genome-wide association studies (GWAS).

### 2.2 Data source

For OSAS, we utilized the published GWAS summary statistics from the FinnGen study, which includes 217,955 European patients (22). FVC and FEV1 data were extracted from UKB. The sample size for FVC (GWAS ID: ukb-b-7953) was 421,986 and for FEV1 (GWAS ID: ukb-b-19657) was also 421,986. The summary statistics for GWAS of diabetic microvascular complications were taken from FinnGen (https://r5.finngen.fi/). DR (GWAS ID: finn-b-DM_RETINOPATHY_EXMORE) analysis involved 14,584 cases and 176,010 controls. BDR is an early stage of DR. The analysis for BDR (GWAS ID: finn-b-DM_BCKGRND_RETINA) included 2,026 cases and 204,208 controls; PDR (GWAS ID: finn-b-DM_RETINA_PROLIF) consisted of 8,681 cases and 204,208 controls. DKD (GWAS ID: finn-b-DM_NEPHROPATHY_EXMORE) had 3,283 cases and 181,704 controls. DN (GWAS ID: finn-b-DM_NEUROPATHY) included 1,415 cases and 162,201 controls. The diagnoses for these conditions are based on their respective International Classification of Diseases(ICD) codes, and we have meticulously organized detailed inclusion and exclusion criteria for each study mentioned above. See Supplementary Materials Tables S1–S3 for details.

### 2.3 Instrument variable selection

Single nucleotide polymorphisms (SNPs) were selected based on the following criteria: (1) SNPs are strongly associated with exposure and reach genome-wide significance (*P* < 5 × 10^−7^); SNPs were not associated with any potential confounders and were independent of each other to avoid bias caused by linkage disequilibrium (*r*^2^ < 0.0001, clustering distance = 10,000 kb); (2) SNPs are associated with outcomes only through exposure. F statistics ($F=\frac{R^{2}\times (N-2)}{1-R^{2}}$, $R^{2}\;(\mathrm{SNP}<10)=2\times EAF\times (1-EAF)\times beta^{2}$, $R^{2}\;(\mathrm{SNP}\geq 10)=$ $\frac{2\times EAF\times (1-EAF)\times beta^{2}}{\left(2\times EAF\times (1-EAF)\times beta^{2})+(2\times EAF\times (1-EAF)\times N\times SE^{2}\right)}$); SNP exposure-associated beta (*β*); variance (SE)). Since an empirical threshold above 10 indicates that the SNP has sufficient validity, SNPs with F statistics less than 10 were removed. We provide information on F statistics, SNPs using the supplementary datasheet. Details of the screened SNP are provided in Supplementary Materials Tables S4–S11.

### 2.4 Data analysis

Linkage Disequilibrium Score Regression (LDSC) analysis is a new method for estimating genetic correlations that require only GWAS summary statistics. Even if there are overlapping individuals between the two GWAS, the regression slope of LDSC provides an unbiased estimate of the genetic correlation (23). LDSC analysis in this study was used to evaluate the genetic correlation of OSAS, FVC, FEV1, and diabetic microvascular disease. First, it is used to reformat summary statistics and remove non-SNP variants (such as indels), chain-ambiguous SNPs, and duplicate SNPs. SNPs with imputation quality scores >0.9 and Minor Allele Frequency (MAF) > 0.01 were selected in our study to prevent bias due to variable imputation quality (24). Second, LD scores were estimated using the 1,000 Genomes Project as the linkage disequilibrium reference panel, following standard methods recommended by the developers. Third, we studied the genetic correlation between OSAS, FVC, FEV1, and diabetic microangiopathy using LDSC (https://github.com/bulik/ldsc). And the strict Bonferroni threshold was set at *P* < 0.0033 (0.05/15). However, after the Bonferroni correction, there was no significant correlation. Therefore, we set the candidate threshold of LDSC regression analysis at *P* < 0.0033 and used MR analysis to verify the causal relationship between OSAS, FVC, FEV1, and diabetic microangiopathy.

Two-sample MR is used to analyze the causal relationship between OSAS, FVC, FEV1, and diabetic microvascular complications. In the absence of horizontal pleiotropy, the IVW method can be used as the main method to analyze causal relationships in MR analysis. Before this, we used Cochrane's Q test to assess heterogeneity among IVs. If heterogeneity is detected (*P* < 0.05), the random-effects IVW model provides a more conservative estimate; otherwise, the fixed-effects IVW model will be used (25). Other MR analysis methods, including Weighted mode, MR-Egger regression, Simple mode, and Weighted mode methods (26), can supplement the IVW method and provide wider confidence intervals (27). The IVW method is applicable when horizontal pleiotropy does not exist (28); If the results of the MR analysis are nominally significant (*P* < 0.05), we consider a possible causal relationship between the exposure and the outcome (29). As the basic model of MR analysis, the IVW method shows good robustness and reliability when dealing with pleiotropic effects. It assumes that all genetic variants contribute uniformly to the causal effect, and obtains an overall causal estimate by weighting the average of the causal estimates of different single nucleotide polymorphisms (SNPs) (30). By comprehensively considering the weights of different genetic variants for analysis, the IVW method can more effectively control possible pleiotropic effects and provide relatively reliable causal estimates (31–34). The IVW method is widely used in MR research and has been widely recognized and accepted in academia and scientific research fields. Its universality as a basic model makes it easier for researchers to understand and use. In addition, the IVW method is one of the simplest and most intuitive methods in MR analysis and does not require overly complex statistical models and calculation processes, allowing researchers to perform analysis and result interpretation more quickly (35). When comparing other methods, the Weighted median is similar to IVW, the Weighted mode method assumes that less than 50% of IVs have horizontal pleiotropy (36), but it uses median weighting instead of inverse variance weighting. Weighted median may be more robust to some skewed or outlier data sets, but may control pleiotropy slightly less than IVW in some cases (34). MR-Egger regression assumes that more than 50% of IVs are affected by horizontal pleiotropy, considers the relationship between the impact of genetic variation on exposure and its impact on outcomes, and can detect and correct biases caused by reverse causation (31, 32). However, the MR-Egger method may not be robust enough to strong horizontal skew (37). Simple mode and Weighted mode methods have poor control over pleiotropic effects and are not as robust as IVW and MR-Egger (38, 39).

We conducted a reverse MR analysis between diabetic microvascular complications and OSAS to examine the possibility of a reverse causal relationship. The procedure for the reverse MR analysis was the same as the aforementioned analysis.

We employed several methods to monitor the possible presence of horizontal pleiotropy. Specifically, *P* values from the MR-Egger intercept test and MR pleiotropy residual sum and outlier (MR-PRESSO) global test could be used to assess the presence of horizontal pleiotropy, and *P* < 0.05 was considered statistically significant (32, 40). The MR-PRESSO outlier test can adjust horizontal pleiotropy by detecting and removing outliers (34). Additionally, we performed a leave-one-out analysis on the identified significant results to determine whether the causal role of the MR analysis was due to a single SNP (41).

Multivariable MR extends the capabilities of MR, akin to evaluating the effects of multiple treatments independently within a single randomized control trial (42). In this approach, the genetic instrument need not be exclusively associated with a single risk factor but can instead relate to a set of measured risk factors, while still adhering to equivalent instrumental-variable assumptions (43). This method accommodates multiple genetic variants, which may not necessarily be linked to every exposure in the model, as well as several causally dependent or independent exposures in an instrumental-variable analysis, thereby disentangling the direct causal effect of each risk factor included in the model (42, 44). Consequently, multivariate MR analysis allows the simultaneous consideration of multiple potential confounding factors, aiding researchers in mitigating the interference of these factors with observed associations and enhancing the accuracy of causal inference (45). In this study, possible confounding factors include obesity, elevated BMI, hyperlipidemia, hypercholesterolemia, etc (46–50). As the main method, we employed a robust IVW method with multiplicative random effects (51).

Given that OSAS is a complicated disease and previous studies have revealed that inflammatory factors and hypertension might mediate the development of diabetic microangiopathy (52–55), we performed a mediation MR analysis using the two-step MR method (56). Based on the literature review, we selected 15 variables that may serve as mediators that may lie on the pathway from sleep apnea syndrome to diabetic microangiopathy with available genetic tools from GWAS, including BMI, triglycerides, cholesterol, High-Density Lipoprotein (HDL), Low-Density Lipoprotein(LDL), apolipoprotein A, apolipoprotein B, blood glucose, C-reactive protein, heart rate, sleep duration, heme oxygenase 1 (53, 54, 57). Then, we screened the mediating factors of the relationship between OSAS and diabetic microangiopathy according to the following criteria: (1) There should be a causal relationship between OSAS and the mediator, and the effect of OSAS on the mediator should be unidirectional, because if the mediation analysis between Bidirectionality exists and the validity of the mediation analysis may be affected (24). (2) Regardless of whether OSAS is adjusted for, the causal relationship between mediators and diabetic microvasculopathy always exists; (3) Based on current scientific evidence, in practice, the relationship between OSAS and mediators, and the association between mediators and diabetic microvasculopathy, should be the other way around. Finally, only one mediating factor, blood glucose level, met all criteria and was included in the mediation analysis to evaluate its mediating effect on the causal relationship between OSAS and diabetic microangiopathy. We then conducted a mediation analysis using a two-step approach. In the first step, we calculated the causal effect of OSAS on mediators (*β*_1_), and in the second step, we estimated the causal effect of mediators on diabetic microangiopathy (*β*_2_). The significance of the mediating effects (*β*_1_*β_2_) and the proportion of the mediation effect in the total effect were estimated using the delta method (58).

All statistical analyses were conducted using R version 4.2.1 (R Foundation for Statistical Computing, Vienna, Austria) along with the “TwoSampleMR”, “MendelianRandomization”, and “MRPRESSO” packages.

### 2.5 Ethics

The ethical data used in our study are publicly available pooled data and their analysis does not require ethical approval.

## 3 Results

### 3.1 Linkage disequilibrium score regression

Regression of LD score between OSAS and diabetic microvasculopathy using summary statistics from the FinnGin database. The results showed a moderate genetic correlation between OSAS and diabetic microangiopathy (*r_g _*= 0.142, SE = 0.044, *P* = 0.0011; *r_g_*_ _= 0.414, SE = 0.077, *P* < 0.001; *r_g_*_ _= 0.398, SE = 0.062, *P* < 0.001). For FVC and FEV1 and diabetic microvasculopathy, we used summary statistics from the UKB and Finnish databases, respectively, to calculate genetic correlations. The results showed a significant genetic correlation between FVC and diabetic microangiopathy (*r_g_*_ _= −0.117, SE = 0.016, *P* < 0.001; *r_g_*_ _= −0.205, SE = 0.028, *P* < 0.001; *r_g_*_ _= −0.153, SE = 0.023, *P* < 0.001). The results also showed a genetic correlation between FEV1 and diabetic microangiopathy (*r_g_* = −0.086, SE = 0.023, *P* = 0.0002; *r_g_* = −0.121, SE = 0.040, *P* = 0.0028; *r_g_* = −0.148, SE = 0.032, *P* < 0.001), as shown in Supplementary Materials Table S12.

### 3.2 Causal effects of OSAS, FVC, and FEV1 on DR

**Figure 2:**
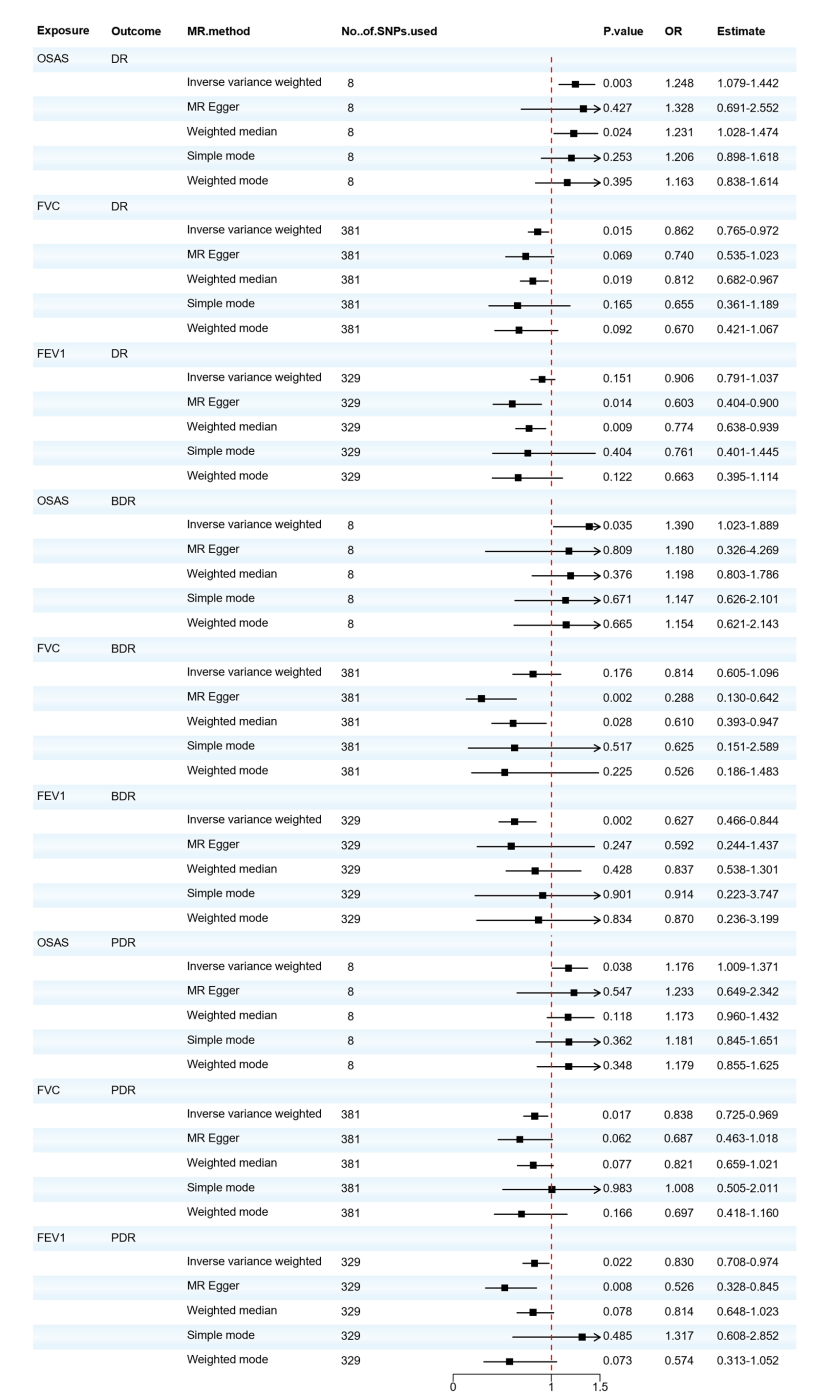
Forest plot of OSAS, FVC, and FEV1 associated with the risk of DR, BDR, and PDR. BDR, background diabetic retinopathy; DR, diabetic retinopathy; FVC, forced vital capacity; FEV1, forced expiratory volume in one second; MR, Mendelian randomization; MR-Eggcr, MR-Egger regression analysis; OSAS, obstructive sleep apnea syndrome; PDR, proliferative diabetic retinopathy; SNP, single nucleotide polymorphism.

Regarding OSAS, as depicted in Figure 2, we observed a potential causal association between OSAS and an increased incidence of DR (OR = 1.248, 95% CI: 1.079–1.442, *P* = 0.003) and also with an increased incidence of BDR and PDR (OR = 1.390, 95% CI: 1.023–1.889, *P* = 0.035; OR = 1.176, 95% CI: 1.009–1.371, *P* = 0.038). In the sensitivity analysis of the IVs, no significant heterogeneity was observed through both the IVW test (Q = 9.703, *P* = 0.206; Q = 2.398, *P* = 0.935; Q = 4.288, *P* = 0.746) and the MR-Egger regression test (Q = 9.643, *P* = 0.141; Q = 2.332, *P* = 0.887; Q = 4.267, *P* = 0.641). The results of the MR–Egger regression analysis indicated that there was no horizontal pleiotropy among the IVs (all *P* > 0.05). The MR-PRESSO test ensured the accuracy of the results (all *P* < 0.05).

We also found that the respiratory-related indicators FVC and FEV1 were positive exposures for diabetic retinopathy, and FVC reduction has a potential causal relationship with the increased incidence of DR and PDR (OR = 0.862, 95% CI: 0.765–0.972, *P* = 0.015; OR = 0.838, 95% CI: 0.725–0.969, *P* = 0.017). There is either no heterogeneity (Q = 423.304, *P* = 0.062; Q = 422.036, *P* = 0.063) or horizontal pleiotropy (*P* > 0.05) in the PDR. Minor heterogeneity could be observed in the DR by IVW testing (Q = 448.273, *P* = 0.009) and MR-Egger testing (Q = 447.105, *P* = 0.009). According to the intercept of MR–Egger regression, it can be found that IVs do not have horizontal pleiotropy (*P* > 0.05), and the MR-PRESSO test ensures the accuracy of the results (all *P* < 0.05). Reduced FEV1 will also increase the risk of BDR and PDR (OR = 0.627, 95% CI: 0.466–0.844, *P* = 0.002; OR = 0.830, 95% CI: 0.708–0.974, *P* = 0.022). There is either no heterogeneity (Q = 321.439, *P* = 0.592; Q = 321.420, *P* = 0.577) or horizontal pleiotropy (*P* > 0.05) in the BDR. However, minor heterogeneity could be observed in the PDR by IVW testing (Q = 377.673, *P* = 0.030) and MR-Egger testing (Q = 373.099, *P* = 0.040). Based on the leave-one-out analysis, no SNP significantly altered the overall results, and the MR-PRESSO test ensures the accuracy of the results (all *P* < 0.05). The MVMR analysis was conducted to assess the direct effect of OSAS on DR with the adjustment of multiple other risk factors for diabetic complications. The results obtained from the two-sample univariable MR analysis were consistent with the findings from the MVMR, but the associations of OSAS with BDR and PDR were no longer significant, as shown in Supplementary Materials Table S14. Detailed results of the MR analysis are presented in Supplementary Materials Tables S13, S15 and the sensitivity analysis results are shown in Supplementary Materials Table S15. Based on the recent MR analysis, we infer that patients with OSAS exhibit an elevated risk for DR development. Individuals with pulmonary function abnormalities are advised to undergo periodic lung function evaluations and implement preventive measures against DR.

### 3.3 Causal effects of OSAS, FVC, and FEV1 on DKD

**Figure 3:**
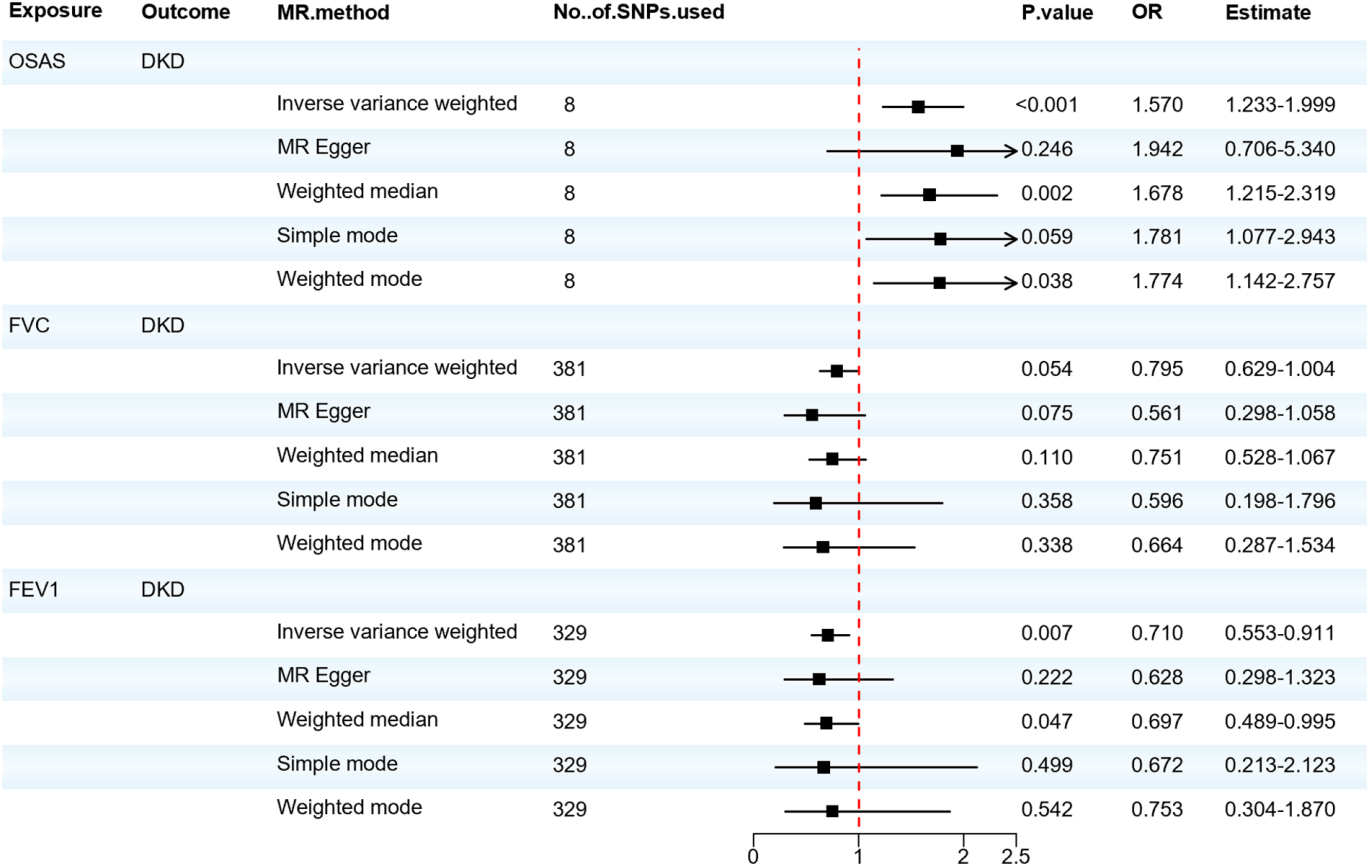
Forest plot of OSAS, FVC, FEV1 associated with the risk of DKD. DKD, diabetic kidney disease; FVC, forced vital capacity; FEV1, forced expiratory volume in one second; MR, Mendelian randomization; MR-Eggcr, MR-Egger regression analysis; OSAS, obstructive sleep apnea syndrome; SNP, single nucleotide polymorphism.

Next, as shown in Figure 3, we evaluated the causal relationship between OSAS and DKD. IVW, Weighted median, and Weighted mode analyses indicated that genetically predicted OSAS is associated with a higher risk of DKD (OR = 1.570, 95% CI: 1.233–1.999, *P* < 0.001; OR=1.678, 95% CI: 1.215–2.319, *P* = 0.002; OR = 1.774, 95% CI: 1.142–2.757, *P* = 0.038). Both the IVW test (Q = 5.853, *P* = 0.557) and the MR-Egger regression test (Q = 5.673, *P* = 0.461) showed no evident heterogeneity. The MR-Egger regression results suggested that there's no horizontal pleiotropy in the IVs (*P* > 0.05), and the MR-PRESSO test confirmed the accuracy of the results (*P* < 0.05). There was no significant correlation between the decrease in FVC and DKD. However, a decrease in FEV1 showed a significant correlation with DKD (OR = 0.710, 95% CI: 0.553–0.911, *P* = 0.007). Although there was slight heterogeneity, our MR–Egger regression results indicated that there was no horizontal pleiotropy in the IVs (*P* > 0.05). The MR-PRESSO test also proved the accuracy of the results (*P* < 0.05), and no SNP significantly altered the overall results, so our results are relatively robust. After adjusting for possible confounding factors including obesity, elevated BMI, hyperlipidemia and hypercholesterolemia, OSAS was still associated with DKD, as shown in Supplementary Materials Table S14. The detailed results of the MR analysis are presented in Supplementary Materials Table S13 and the results of the sensitivity analysis are shown in Supplementary Materials Table S15. This suggests that the FEV1 level plays a pivotal role in the pathogenesis of DKD. Concurrently, patients with OSAS should be vigilant in taking preventive measures against the onset of DKD.

### 3.4 Causal effects of OSAS, FVC, and FEV1 on DN

**Figure 4:**
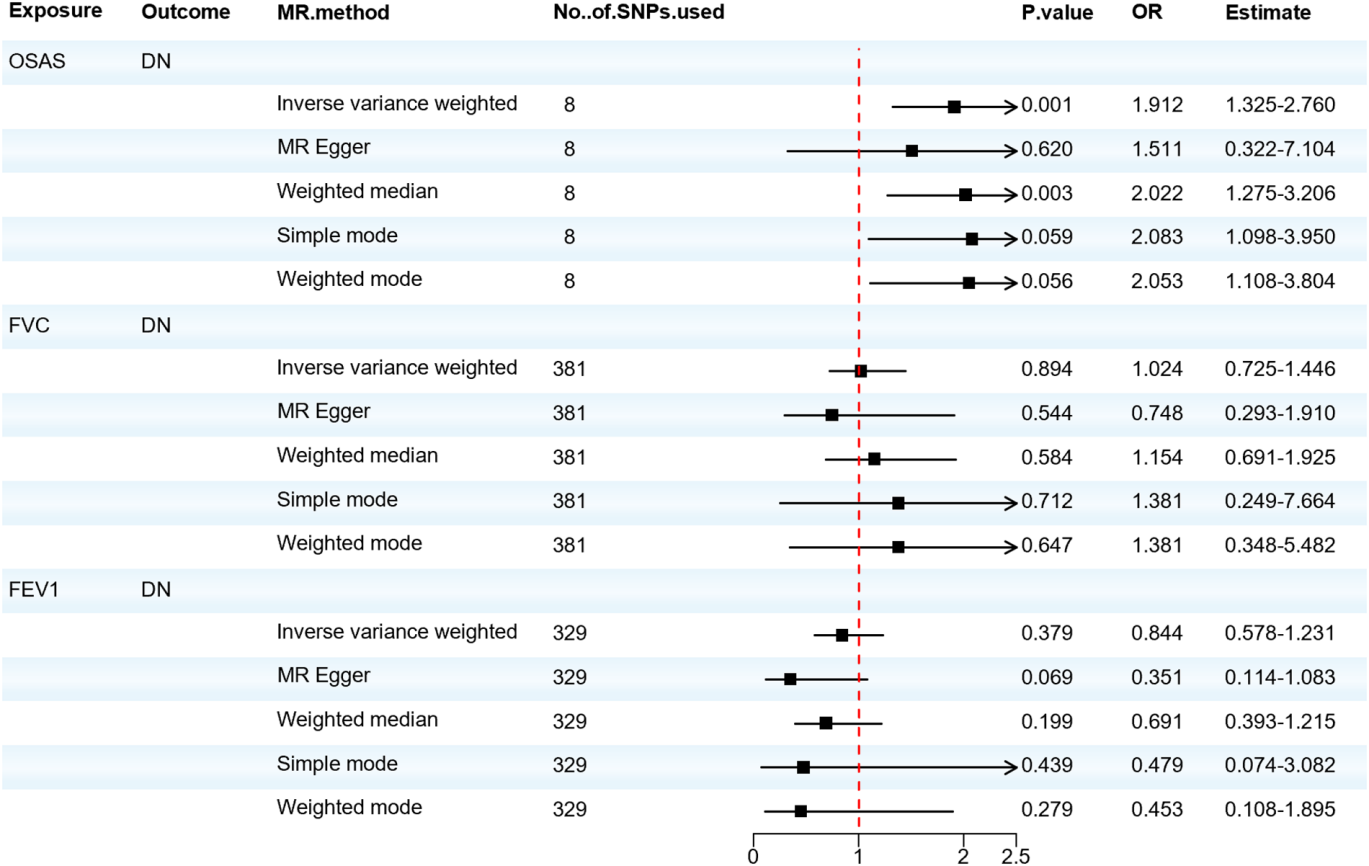
Forest plot of OSAS, FVC, FEV1 associated with the risk of DN. DN, diabetic neuropathy; MR, Mendelian randomization; MR-Eggcr, MR-Egger regression analysis; FVC, forced vital capacity; FEV1, forced expiratory volume in one second; OSAS, obstructive sleep apnea syndrome; SNP, single nucleotide polymorphism.

We also analyzed the causal relationship between OSAS and DN. The IVW and Weighted median analyses indicated that genetically predicted OSAS is associated with a high risk of DN (OR = 1.912, 95% CI: 1.325–2.760, *P* = 0.001). Both the IVW test (Q = 1.141, *P* = 0.992) and the MR-Egger regression test (Q = 1.047, *P* = 0.984) showed that there is no heterogeneity among the IVs. The MR-Egger regression revealed no horizontal pleiotropy for the IVs (*P* > 0.05). However, there was no significant association between the risk of DN and either FVC or FEV1. As shown in Figure 4. OSAS was still associated with DN after adjusting for possible confounding factors such as obesity, elevated BMI, hyperlipidemia, and hypercholesterolemia, as shown in Supplementary Materials Table S14. The results of the sensitivity analysis can be found in Supplementary Materials Table S15. This reminds us that attention should be paid to the possibility of developing DN when patients with OSAS.

### 3.5 Causal effects of diabetic microvascular complications on OSAS

**Figure 5:**
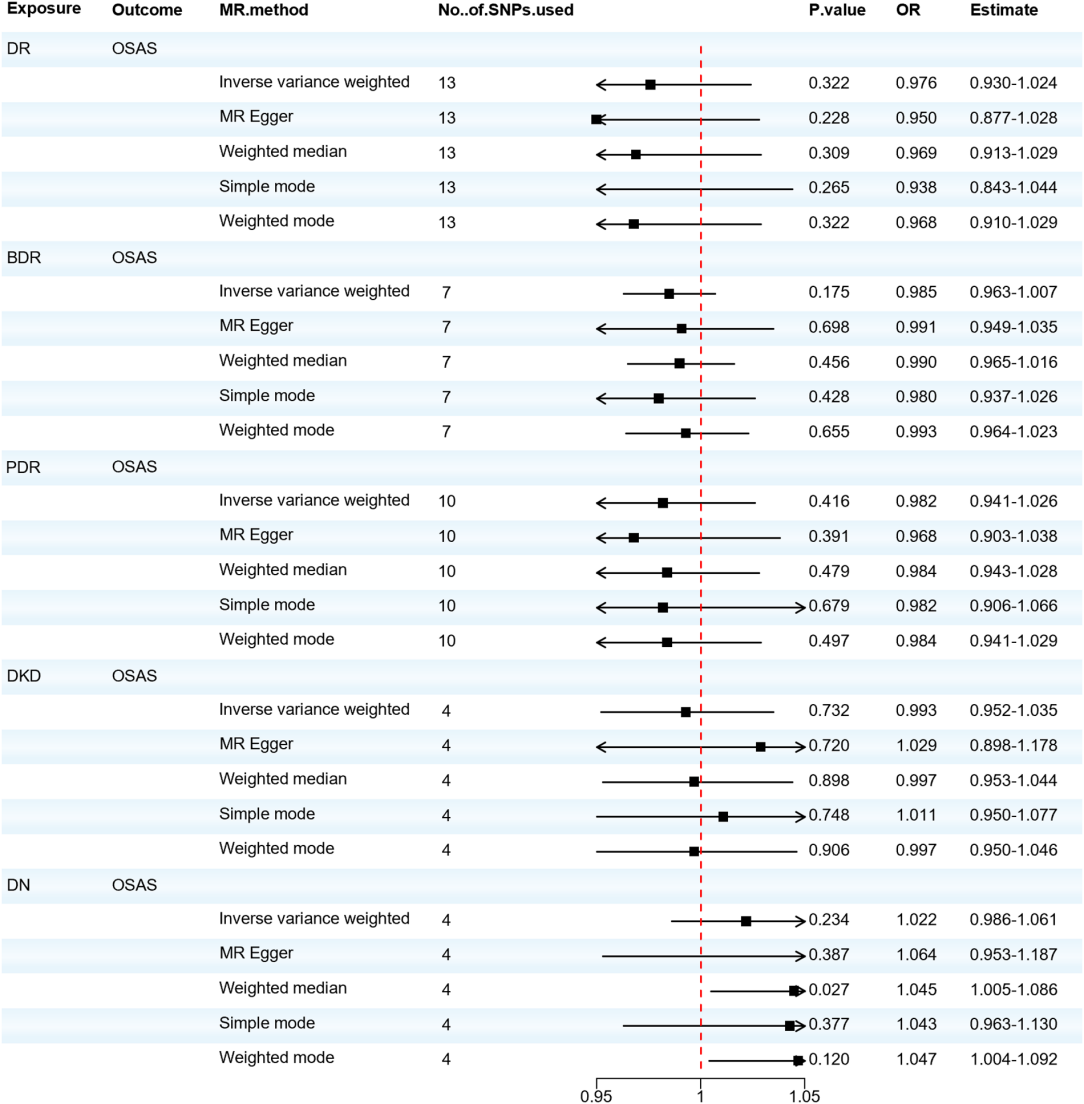
Forest plot of DR, BDR, PDR, DKD, DN associated with the risk of OSAS. BDR, background diabetic retinopathy; DKD, diabetic kidney disease; DN, diabetic neuropathy; DR, diabetic retinopathy; MR, Mendelian randomization; MR-Eggcr, MR-Egger regression analysis; PDR, proliferative diabetic retinopathy; OSAS, obstructive sleep apnea syndrome; SNP, single nucleotide polymorphism.

When considering OSAS as the outcome, we found no significant association between DR, DKD, and the risk of developing OSAS. Although the Weighted median analysis suggested that DN might increase the risk of OSAS (OR = 1.045; 95% CI: 1.005–1.086; *P* = 0.027), our primary analysis method, the IVW analysis, indicated no significant association between DN and OSAS (OR = 1.022; 95% CI: 0.986–1.061; *P* = 0.234). The IVW test (Q = 4.459, *P* = 0.216) and the MR-Egger test (Q = 3.479, *P* = 0.176) did not observe significant heterogeneity. The MR-Egger regression analysis indicated no horizontal pleiotropy between the exposure and the outcome (*P* > 0.05). Thus, the reverse MR suggests there's no significant association between diabetic microvascular complications and OSAS, as shown in Figure 5. Furthermore, we found that there's no reverse causal relationship between FVC and diabetic microvascular complications. In the IVW analysis, when using FEV1 as the outcome, there was a reverse causal relationship between FEV1 and DR, BDR, and PDR (OR = 0.981; 95% CI: 0.966–0.997; *P* = 0.021; OR = 0.990; 95% CI: 0.980–0.999; *P* = 0.029; OR = 0.984; 95% CI: 0.972–0.998; *P* = 0.020), as shown in Supplementary Materials Table S16. The results of the sensitivity analysis can be found in Supplementary Materials Table S17. Subsequently, we performed a mediation MR analysis, and unfortunately, in the current sample and data conditions, we were unable to determine that blood glucose levels played a significant mediator between OSAS and diabetic microangiopathy. This finding suggests that a larger sample size or finer statistical methods may be needed to further explore this mediation effect, as shown in Supplementary Materials Table S18.

## 4 Discussion

Our study found that a genetic correlation between OSAS, FVC, FEV1 and diabetic microangiopathy exists and genetically predicted OSAS leads to an increased incidence of diabetic microangiopathy. Among them, the impact of OSAS on DN is the most significant. Patients with OSAS have a risk of developing DN that is 1.91 times that of normal individuals, while the risks of developing DR and DKD for such patients are 1.25 and 1.57 times respectively compared to normal individuals. In terms of DR, the effect of OSAS on BDR is more pronounced than on PDR. Furthermore, the lung function indicators FVC and FEV1 are protective factors against diabetic microangiopathy, and a bidirectional causality exists between FEV1 and DR, BDR and PDR. After adjusting for possible confounding factors such as obesity, elevated BMI, hyperlipidemia, and hypercholesterolemia using MVMR, OSAS is still associated with DR, DKD, and DN, but the association with BDR and PDR is no longer significant. The results of the LDSC analysis also showed a genetic correlation between OSAS and DR, but this correlation was no longer significant in the BDR and PDR. It may be because BDR and PDR are specific subtypes of DR. It may have different risk factors or causal pathways than DR overall. The genetic variants used in analyses may capture the causal effects of BDR and PDR less effectively than DR overall. In addition, as subgroups of DR, BDR, and PDR have smaller samplesizes than DR, the smaller sample sizes may result in reduced statistical power to detect true associations, which may explain the loss of significance (59, 60).

The application of pulmonary function testing in OSAS patients is for a comprehensive evaluation of the respiratory system (61). Although the diagnosis of OSAS mainly relies on polysomnography (PSG), pulmonary function testing is of great significance for evaluating the patient's respiratory function status (30). There is literature showing that FEV1 and FVC in pulmonary function tests are related to the severity of OSAS, which reflects the ventilatory dysfunction that occurs in OSAS patients during sleep and its impact on the respiratory system (62). In addition, through pulmonary function testing, we can evaluate the patient's vital capacity, ventilatory function, airflow limitation, and other indicators (63). These indicators can provide a comprehensive understanding of the respiratory function status of OSAS patients (30, 64). In clinical practice, pulmonary function test results can not only guide the selection of treatment strategies. For example, OSAS patients with abnormal pulmonary function may require different treatment options, such as continuous positive airway pressure (CPAP) therapy or physical exercise, but also Treatment effects and changes in condition can be monitored (30). Therefore, pulmonary function testing plays an important role in the management of OSAS. It can not only help doctors comprehensively evaluate the patient's respiratory function, but also guide the selection and adjustment of treatment options to ensure that the patient's respiratory function is optimally controlled and managed.

Diabetic microangiopathy represents hallmark manifestations of the chronic progression of diabetes. During the non-proliferative phase, DR manifests as microaneurysms and retinal hemorrhages. As it progresses to the proliferative phase, ischemia or edema in the macular region, vitreous hemorrhage, and tractional or rhegmatogenous retinal detachment can lead to significant visual impairment, even blindness (65). DKD is characterized by proteinuria and a decline in glomerular filtration rate (66), often advancing to uremia (67). DN is a complex neurologic disorder affecting both peripheral and autonomic nervous systems. Symptoms may include pain, numbness, balance issues, and foot ulcers (2, 68, 69), increasing the risk of diabetic foot and amputation. In summary, diabetic microangiopathy can impair bodily functions, potentially leading to disability, and reducing employment opportunities and the work capacity of patients (70, 71).

In previous observational studies, Chang and colleagues found an association between the presence and severity of OSAS and DR (72). After adjusting for all possible confounding factors, Tahrani et al. found that OSAS remained an independent risk factor for Diabetic Peripheral Neuropathy (DPN) (73). Leong et al. conducted a comprehensive analysis of 2 longitudinal and 10 cross-sectional studies. Multivariate analysis indicated a significant correlation between OSAS and DKD, which was confirmed through a meta-analysis of another 7 studies (74). Furthermore, Ouardighi et al. compared the prevalence of OSAS in patients with diabetic microangiopathy and assessed the potential effects of diabetic microangiopathy on OSAS. The results showed no correlation between diabetic microangiopathy and OSAS (75). Hsin-Chieh et al. found that a decrease in FVC and FEV1 could increase the risk of diabetes, and chronic hyperglycemia and tissue hypoxia could promote the onset of microangiopathy (76). Our bidirectional MR study further supplements previous research and provides evidence for the potential causal relationship between OSAS and diabetic microangiopathy.

The development of diabetic microvascular complications may be attributed to OSAS and its associated cyclical drops in oxygen saturation and disruptions in sleep structure. This leads to several biological changes, including the activation of ADP-ribose polymerase, protein kinase C, and the polyol pathway. Additionally, there's an increase in the production of advanced glycation end-products, oxidative and nitrosative stress, as well as the activation of the sympathetic nervous system and the renin-angiotensin-aldosterone system (RAAS) (11, 77). All these biological changes can lead to endothelial dysfunction, triggering inflammatory responses and cell apoptosis, resulting in damage to the vascular wall, increased permeability, white blood cell infiltration, and cell death. These conditions stimulate the production of hypoxia-inducible factors, leading to an increased expression of vascular endothelial growth factor (VEGF) and a higher rate of neovascularization. These factors collectively contribute to the progression of diabetic microvascular complications (73, 78, 79). Additionally, ventilation abnormalities can lead to decreased FVC and dynamic lung compliance (80), the decline in lung function, leading to a cumulative loss of pulmonary reserves, ultimately exacerbates tissue hypoxia associated with vascular lesions in distant organs. This is the fundamental cause of diabetic microvascular complications (81). At the same time, reductions in FVC and FEV1 are correlated with increased levels of Hypoxia-Inducible Factor-1 (HIF-1) and VEGF (51), thereby heightening the likelihood of endothelial vascular lesions related to diabetes.

From a clinical perspective, as the first MR study on the role of OSAS in the etiology of diabetic microangiopathy, this study suggests that the genetic susceptibility to OSAS may account for variations in diabetic microangiopathy in people of European descent. Although OSAS has a genetic component, it is also influenced by environmental and lifestyle factors and is possibly preventable (82). Although there is still uncertainty about the exact functions of the 8 SNPs, their polygenic effects on diabetic microvascular complications, and the mechanisms by which these gene variants operate, current evidence still suggests that reduced blood oxygen saturation plays a significant role in diabetic microvascular complications. It seems prudent to recommend that people at high risk of diabetic microangiopathy strengthen the management of OSAS and take measures including lifestyle changes by strengthening social publicity and education and improving residents' health awareness. In addition, detecting self-oxygen saturation, monitoring pulmonary function parameters, and timely adjustment for abnormal pulmonary function seem to have unexpected effects on the prevention of clinical DR and its subtypes. Future work should try to clarify potential mechanisms, aiming to intervene, provide information for public health research, or further enhance our understanding of the etiology of diabetic microvascular complications. Instrumental variable SNPs can be incorporated as genetic predictors in predictive models aimed at identifying populations most likely to benefit from specific interventions. In our study, there were 8 SNPs for diabetic microangiopathy as instrumental variables for OSAS. Future research could consider building predictive models based on these SNPs and validating them in longitudinal cohorts for early detection and intervention in individuals at risk of diabetic microvascular complications. As our understanding of human genetics and the interactions between genes, metabolomics, proteomics, and transcriptomics grows, future MR studies should integrate these aspects to identify new biomarkers predicting the onset of diabetic microvascular complications and screen potential therapeutic targets. By screening SNP-related protein factors as instrumental variables to estimate the causal impact of this protein factor on specific results, MR studies can be used to assess whether the drug is likely to be effective in the study of compounds targeting specific proteins, guiding clinical decision-making and treatment planning. Lastly, in this study, we adopted a comprehensive dataset derived from public databases encompassing cases of microvascular complications induced by both Type 1 diabetes mellitus (T1DM) and Type 2 diabetes mellitus (T2DM). Given that sustained hyperglycemic conditions in both types of diabetes can trigger a series of intricate metabolic and molecular cascades, leading to endothelial dysfunction within the microvasculature, this constitutes the primary pathological mechanism (83). We posit that merging and analyzing datasets from T1DM and T2DM collectively, as opposed to separate analyses, significantly enhances the statistical power of the study. This analytical strategy aids in uncovering universal characteristics of DR across the entire diabetic population, rather than focusing solely on specific diabetic subtypes (84). Furthermore, through the amalgamation analysis, we can circumvent potential issues of reduced statistical significance due to insufficient sample sizes, thereby enhancing the generalizability and credibility of study findings (85). The central objective of this study is to capture the overall trends of DR, rather than distinguishing between specific subtypes of diabetes. Through this comprehensive analytical approach, we aim to provide a more thorough and profound scientific basis for the prevention and therapeutic intervention of microvascular complications in diabetes.

Our current research has several advantages. Firstly, few studies have comprehensively investigated the relationship between OSAS and the incidence of diabetic microvascular complications. We are the first to examine their potential causal relationship using the MR method and a large amount of GWAS data. Secondly, because we used a two-sample MR analysis, our results are less likely to be confounded and reverse causality compared to traditional observational studies. In addition, we utilized large-scale samples to improve the statistical power of the study and make the findings more convincing. MR designs estimate the causal effects of independent variables on dependent ones rather than merely observing their correlation. Thus, the advantages of MR analysis may enhance the reliability of our findings, provide stronger evidence for clinical decision-making, and assist doctors and patients in making more informed medical choices.

However, some limitations were identified in our study. First, MR requires three strict core assumptions to be met: relevance, independence, and exclusion restriction. While we employed a rigorous study design to avoid violating these assumptions and identified closely related genetic tools for exposure (*P*-value < 5 × 10^−7^) with F-statistics > 10, and replicated results with multiple sensitivity analyses. Additionally, we used MR-Egger to identify potential horizontal pleiotropy, but it's impossible to completely rule out residual pleiotropy. In our study, no horizontal pleiotropy was found between OSAS with diabetic microvascular complications. However, horizontal pleiotropy exists between FEV1 and PDR, indicating that their relationship might be influenced by pleiotropic factors and warrants further validation. Second, there may be some sample overlap in our study, the direction and extent of any bias remain uncertain. Recent simulation studies also suggest that two-sample MR methods can be safely applied to single-sample MR performed in large biobanks. Hence, any bias due to sample overlap, if present, might be minimal (82). Third, clinical trials typically assess short-term intervention effects over shorter durations. This implies that our findings might not provide information about short-term intervention effects, which could be crucial for questions directly related to clinical interventions. Although our research can reveal the relationship between OSAS and DR, the application of the findings in real-world clinical settings might require additional research to determine whether treatment or interventions for OSAS are needed and how they should be conducted. Lastly, since the UK Biobank represents a biased sample of healthy older individuals from the UK, the Finnish database population also has specific demographic characteristics, including genetic background, genetic diversity, lifestyle, dietary habits, and genomic features, among others. These characteristics might differ from those of other countries or populations, making the research findings potentially inapplicable to other groups (86). Due to the lack of individual-level data, it's not possible to evaluate the relationship between the severity of OSAS and other parameters. Therefore, our findings should be interpreted with caution and validated in further studies.

## 5 Conclusion

Our study offers suggestive causal evidence indicating a potential causal relationship between OSAS and diabetic microvascular complications. Lung function might also be associated with the risk of diabetic microvascular disease onset. Our findings suggest that lifestyle interventions related to OSAS could serve as preventive strategies for potential populations at risk of diabetic microvascular complications. Our research is comprehensive and lays the groundwork for further large-scale longitudinal studies or randomized controlled trials

## Data Availability

Publicly available datasets were analyzed in this study. This data can be found here: https://gwas.mrcieu.ac.uk/.
